# Passage of an Anterior Odontoid Screw through Gastrointestinal Tract

**DOI:** 10.1155/2017/2923696

**Published:** 2017-01-17

**Authors:** L. Leitner, C. I. Brückmann, M. M. Gilg, G. Bratschitsch, P. Sadoghi, A. Leithner, R. Radl

**Affiliations:** Department of Orthopaedic Surgery, Medical University of Graz, Graz, Austria

## Abstract

*Purpose*. Anterior screw fixation has become a popular surgical treatment method for instable odontoid fractures. Screw loosening and migration are a rare, severe complication following anterior odontoid fixation, which can lead to esophagus perforation and requires revision operation.* Methods*. We report a case of screw loosening and migration after anterior odontoid fixation, which perforated the esophagus and was excreted without complications in a 78-year-old male patient.* Results*. A ventral dislocated anterior screw perforated through the esophagus after eight years after implantation and was excreted through the gastrointestinal (GI) tract. At a 6-month follow-up after the event the patient was asymptomatic.* Conclusion*. Extrusion via the GI tract is not safe enough to be considered as a treatment option for loosened screws. Some improvements could be implemented to prevent such an incident. Furthermore, this case is a fine example that recent preoperative imaging is mandatory before revision surgery for screw loosening.

## 1. Introduction

Odontoid fractures are the most common type of cervical spine fractures in patients older than 70 years [1] often resulting from high energy trauma including falling from a substantial height with hyperextension [2, 3]. Amongst odontoid fractures, type II fractures according to Anderson and d'Allonso classification account for up to 74% [4]. Type II fractures go through the base of the dens and are considered unstable, since a high rate of nonunion up to 30% has been described [5, 6]. Treatment options for type II fractures of the odontoid consist of conservative treatment with a rigid or halo brace, which is in general correlated with poor functional outcome and increased mortality [7], or surgical treatment using either an anterior odontoid screw placement or posterior fixation techniques [8, 9]. Whilst anterior odontoid fixation preserves atlantoaxial motion, in elderly patients posterior C1-C2 fusion seems to lead to the best clinical results and low nonunion rate [10]. Based on a recent review operative treatment is recommended for type II odontoid fractures in the elderly population (>65 years) [11].

The present case report describes a 78-year-old patient who presented with dysphagia eight years following anterior odontoid screw fixation. The odontoid screw appeared to be dislocated ventrally and passed through the gastrointestinal tract in the further course, from where it was excreted without complications.

## 2. Case Report

**Figure 1:**
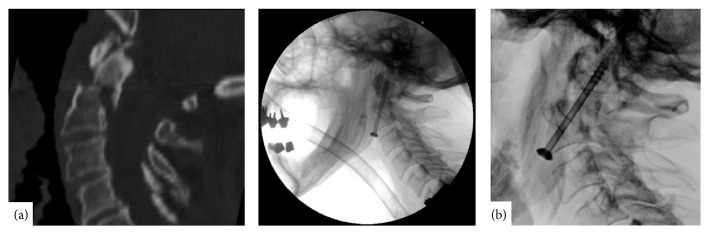
(a) Preoperative computed tomography (CT) of the odontoid fracture, sagittal view (left). Intraoperative view on odontoid screw placement, lateral view (right). (b) X-ray imaging disclosed a five-millimeter dorsal migration of the odontoid process three weeks after the operation, lateral view.

**Figure 2:**
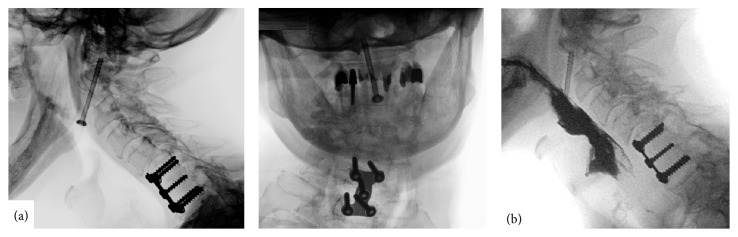
(a) X-ray imaging disclosed a ventral migration of the odontoid screw, 8 years after operation, lateral view (left) and a.p. view (right). (b) Pharyngeal stenosis was excluded by fluoroscopic swallow examination, lateral view.

**Figure 3:**
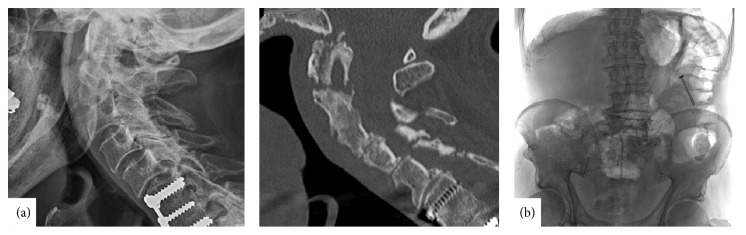
(a) X-ray (left) and computed tomography (CT) (right) show missing odontoid screw and not consolidated fracture gap, sagittal view. (b) Abdomen X-rays displaying an odontoid screw in the left upper abdomen, presumably descendent colon, with no signs of free peritoneal air, a.p. view.

A 78-year-old man, with a history of anterior screw fixation of an odontoid type II fracture after falling from a two-meter height eight years ago (Figure 1(a)), presented with newly occurred dysphagia. Additionally, ventral fusion of C6/C7 to C7/Th1 had been performed after traumatic fracture 32 years ago (visible on Figures 2 and 3). Anterior odontoid screw placement had been chosen as less invasive method due to concomitant cardiac insufficiency with valvular defects and advanced stage of coronary heart disease. The operation report states that position of the cannulated spongiosa screw had to be changed once but was strongly fixated. It is likely that the odontoid fracture was not reduced well with this surgery technique, as a five-millimeter dorsal migration of the odontoid process three weeks after the operation (Figure 1(b)) led to halo fixator installation, which had to be refixed once, for 16 weeks. The patient did not continue follow-up until dysphagia occurred.

X-ray imaging of the symptomatic patient eight years after operation disclosed a ventral migration of the odontoid screw (Figure 2(a)). Subsequent magnetic resonance imaging (MRI) revealed dorsal abduction of the odontoid process with the screw head reaching into the prevertebral soft tissues with unclear distention. Pharyngeal stenosis was excluded by fluoroscopic swallow examination (Figure 2(b)). The patient was neurologically asymptomatic besides complaining about neck tension, and he was further mobilized with a foam cervical collar. Elective operative screw removal via a ventral approach was scheduled.

When the patient was hospitalized in our center for revision surgery, four weeks after the migration was first diagnosed, a preoperative planning computed tomography (CT) was performed. The screw could not be detected anymore, the eight-year-old fracture gap was not consolidated, and the odontoid process was not dislocated (Figure 3(a)). Immediately performed chest and abdomen X-rays displayed the missing screw in the left upper abdomen, presumably descendent colon, without signs of free peritoneal air (Figure 3(b)). Otorhinolaryngologic examination described a granulated pharyngeal spot without infection signs. Two days later the screw was excreted noteless.

## 3. Treatment

Prophylactic antibiotic therapy with amoxicillin/clavulanic acid for seven days was initiated when passage of the screw into the gastrointestinal system became evident. The patient was discharged after excretion of the screw, and full mobilization and gradual weaning from the foam cervical collar were advised. Surgical odontoid refixation was not considered due to the concomitant diseases as described above and lack of symptoms. At a 6-month follow-up after the event the patient was asymptomatic.

## 4. Discussion

In a meta-analysis of 48 studies on anterior odontoid screw fixation a random-effect pooled reoperation rate of 5% was calculated caused by heterogeneous reasons including device related reasons as screw loosening or migration and screw-pullout [12]. Screw loosening and consequent pharyngeal perforation are an uncommon, but well recognized, occurrence for ventral cervical spine fusion operations in literature [13–16]. Although this complication has also been described after odontoid fixation [17, 18], to the best of our knowledge this is the first case of transpharyngeal odontoid screw migration and spontaneous resolution via deglutition.

The clinical presentation of oesophageal perforation can range from asymptomatic presentation to local infection to infection of the mediastinum and death and therefore is considered a severe complication [15, 16]. Since available literature suggests operative treatment of type II fractures in elderly (>65 years) population [7, 10], case numbers are increasing and odontoid screw migration is likely to become a more frequent incident.

According to earlier reports of screw and plate loosening following cervical spine fusion, initial inadequate positioning or engagement of screws in a plate and local infection seems to be the main cause for this incident [18, 19]. In the present case, the position of the screw had to be changed once, and a five-millimeter dorsal migration of the odontoid process occurred three weeks after surgery, suggesting a suboptimal holding of the screw (Figure 1(b)). If this event could have been prevented with an intraoperative switch to a two-screw anterior fixation technique cannot be answered yet, and whether one- or two-screw anterior fusion technique is more appropriate is still a subject of debate [20]. When the migration was diagnosed, a halo fixator was installed for 16 weeks as an attempt of secondary conservative fixation. The fracture line was still visible after 8 years (Figure 3(a)), indicating that there was a nonunion which can also cause loosening and migration of the screw. Some authors suggest posterior screw fixation as a salvage procedure when anterior fixation fails [21]; the patient's denial and his health status present a contraindication for repeated surgery.

We report a time delay of four weeks between the first radiologic diagnosis of screw migration and hospitalization for screw extraction. Esophageal perforation could have been avoided by rapid surgery, as suggested by other published cases of screw migration of the cervical region [19].

## 5. Conclusion

Even though this incident did not lead to serious injury of the patient, we considered it a serious device caused complication worth publication. Extrusion via the GI tract is not safe enough to be considered as a treatment option; some improvements could be implemented to prevent such an incident: (1) the most important lesson to be learned from this case is that surgery for revision should be scheduled as soon as possible when screw loosening or migration appears in the neck region, to prevent potentially fatal complications. (2) The patient discontinued radiological follow-up one year after his operation, which should have been extended in synopsis with the initially not fully satisfying surgical result. Monitoring of the fracture healing using postoperative CT examination could have led to earlier intervention. (3) The present case is a fine example that recent preoperative imaging is mandatory before revision surgeries of loosen screws in the cervical spine region.

## References

[1] Elgafy H., Dvorak M. F., Vaccaro A. R., Ebraheim N. (2009). Treatment of displaced type II odontoid fractures in elderly patients. *American journal of orthopedics (Belle Mead, N.J.)*.

[2] Ivancic P. C. (2014). Odontoid fracture biomechanics. *Spine*.

[3] Alker G. J., Oh Y. S., Leslie E. V., Lehotay J., Panaro V. A., Eschner E. G. (1975). Postmortem radiology of head and neck injuries in fatal traffic accidents. *Radiology*.

[4] Clark C. R., White A. A. (1985). Fractures of the dens. A multicenter study. *Journal of Bone and Joint Surgery A*.

[5] Anderson L. D., D'Alonzo R. T. (1974). Fractures of the odontoid process of the axis. *The Journal of Bone & Joint Surgery—American Volume*.

[6] Berlemann U., Schwarzenbach O. (1997). Dens fractures in the elderly. Results of anterior screw fixation in 19 elderly patients. *Acta Orthopaedica Scandinavica*.

[7] Hanigan W. C., Powell F. C., Elwood P. W., Henderson J. P. (1993). Odontoid fractures in elderly patients. *Journal of Neurosurgery*.

[8] Boehler J. (1982). Anterior stabilization for acute fractures and non-unions of the dens. *Journal of Bone and Joint Surgery A*.

[9] Dickman C. A., Sonntag V. K. H., Papadopoulos S. M., Hadley M. N. (1991). The interspinous method of posterior atlantoaxial arthrodesis. *Journal of Neurosurgery*.

[10] Scheyerer M. J., Zimmermann S. M., Simmen H.-P., Wanner G. A., Werner C. M. L. (2013). Treatment modality in type II odontoid fractures defines the outcome in elderly patients. *BMC Surgery*.

[11] Harrop J. S., Hart R., Anderson P. A. (2010). Optimal treatment for odontoid fractures in the elderly. *Spine*.

[12] Tian N.-F., Hu X.-Q., Wu L.-J. (2014). Pooled analysis of non-union, re-operation, infection, and approach related complications after anterior odontoid screw fixation. *PLOS ONE*.

[13] Kim S. B., Oh S. H., Kim Y. S., Ko Y., Chung W. S. (2004). Delayed esophageal perforation after an anterior cervical plating. *Korean Journal of Spine*.

[14] Nourbakhsh A., Garges K. J. (2007). Esophageal perforation with a locking screw: a case report and review of the literature. *Spine*.

[15] Pompili A., Canitano S., Caroli F. (2002). Asymptomatic esophageal perforation caused by late screw migration after anterior cervical plating: report of a case and review of relevant literature. *Spine*.

[16] Yee G. K. H., Terry A. F. (1993). Esophageal penetration by an anterior cervical fixation device: a case report. *Spine*.

[17] Aebi M., Etter C., Coscia M. (1989). Fractures of the odontoid process. Treatment with anterior screw fixation. *Spine*.

[18] Lee E. J., Jang J. W., Choi S. H., Rhim S. C. (2012). Delayed pharyngeal extrusion of an anterior odontoid screw. *Korean Journal of Spine*.

[19] Cagli S., Isik H. S., Zileli M. (2009). Cervical screw missing secondary to delayed esophageal fistula: case report. *Turkish Neurosurgery*.

[20] Feng G., Wendlandt R., Spuck S., Schulz A. P. (2012). One-screw fixation provides similar stability to that of two-screw fixation for type ii dens fractures. *Clinical Orthopaedics and Related Research*.

[21] Joaquim A. F., Patel A. A. (2015). Surgical treatment of Type II odontoid fractures: anterior odontoid screw fixation or posterior cervical instrumented fusion?. *Neurosurgical focus*.

